# Co-delivery of docetaxel and bortezomib based on a targeting nanoplatform for enhancing cancer chemotherapy effects

**DOI:** 10.1080/10717544.2017.1362677

**Published:** 2017-08-08

**Authors:** Junpeng Nie, Wei Cheng, Yunmei Peng, Gan Liu, Yuhan Chen, Xusheng Wang, Chaoyu Liang, Wei Tao, Yinping Wei, Xiaowei Zeng, Lin Mei

**Affiliations:** a School of Life Sciences, Tsinghua University, Beijing, PR China;; b Graduate School at Shenzhen, Tsinghua University, Shenzhen, PR China;; c School of Pharmaceutical Sciences (Shenzhen), Sun Yat-sen University, Guangzhou, PR China;; d Department of Radiation Oncology, Zhongshan Hospital, Fudan University, Shanghai, PR China;; e Brigham and Women’s Hospital, Harvard Medical School, Boston, MA, USA

**Keywords:** Cancer nanotechnology, polydopamine, pH-response, co-delivery, targeting nanoplatform

## Abstract

Using facile polydopamine (PDA)-based surface modification and a pH-sensitive catechol-boronate binding mechanism, a novel drug delivery system was designed for the treatment of breast cancer. The system was able to achieve the following goals: active targeting, pH responsiveness, *in vivo* blood circulation for a prolonged period of time, and dual drug loading. After coating with PDA, the docetaxel (DTX)-loaded star-shaped copolymer cholic acid-poly(lactide-*co*-glycolide) nanoparticles (CA-PLGA@PDA/NPs) were functionalized with amino-poly(ethylene glycol)-folic acid (NH_2_-PEG-FA) and bortezomib (BTZ) to form the targeting composition, DTX-loaded CA-PLGA@PDA-PEG-FA + BTZ/NPs. The novel NPs exhibited similar drug release characteristics compared to unfunctionalized CA-PLGA/NPs. Meanwhile, the incorporated NH_2_-PEG-FA contributed to active targeting which was illustrated by cellular uptake experiments and biodistribution studies. Moreover, the pH responsive binding between BTZ and PDA was demonstrated to be effective to release BTZ at the tumor acidic environment for synergistic action with DTX. Both *in vitro* cytotoxicity and *in vivo* antitumor studies demonstrated that the novel nanoplatform exhibited the most suitable therapeutic effects. Taken together, the versatile PDA modified DTX-loaded CA-PLGA@PDA-PEG-FA + BTZ/NPs offered a promising chemotherapeutic strategy for enhancing breast cancer treatment.

## 1. Introduction

The series of widespread nanotechnology applications with tumor-targeted delivery of known anticancer drugs demonstrate that nanomedicine may significantly enhance drug efficacy and, potentially, effectively reduce drug side effects(Bertrand et al., 2014; Zeng et al., 2017; Zhang et al., 2017). Targeted drug delivery represents an effective strategy for the local accumulation of pharmaceuticals at the tumor sites(Kamaly et al., 2012; Tao et al., 2016; He et al., 2017; Yang et al., 2017). A drug delivery system incorporating active targeting ligands (i.e. saccharides, peptides, antibodies and aptamer) (Ulbrich et al., 2016) may reach preconceived organs, cells or organellas and release the drug load. A series of folic acid (FA)-mediated targeting approaches (Geszke et al., 2011; Wang et al., 2011; Puligujja et al., 2015) based on the high affinity between the folic acid (FA) (a stable, water soluble, poorly immunogenic vitamin B) and the folate receptor (FR) (Porta et al., 2013) have attracted considerable interest in the scientific community. Considering the over-expression of FRs in human cancer cells (including ovarian, kidney, breast, uterus, colon, and lung cancer) and the reduced expression in healthy cells (Au et al., 2016; Zhang et al., 2016), a FA-conjugated drug delivery system may greatly improve the efficacy of recognizing and targeting cancer cells as well as reduce potential damages to healthy cells.

Polymeric nanoparticles (NPs), a verified drug carrier type, have been applied to a variety of nanomedicine formulations due to excellent biodegradation properties(Tao et al., 2013; Feng et al., 2014; Zhang et al., 2014; Zhu et al., 2014; Liu et al., 2017c) exhibited by this material. In our previous studies, we have shown that star-shaped PLGA/NPs feature some advantages over linear PLGA/NPs (Zeng et al., 2013), including a lower solution viscosity, smaller hydrodynamic radius, higher drug loading content (LC), and higher drug encapsulation efficiency. On the basis of these properties, we designed star-shaped polymer CA-PLGA/NPs as a drug delivery vehicle. In view of the fact that active targeting largely depends on passive accumulation based on enhanced permeability and retention (EPR) effects, the formed NPs with active targeting ligands need to exhibit high stability in prolonged blood circulation and further require a proper size for adequate extravasation and tumor accumulation. For the first requirement, a proven process is coating the NPs with a hydrophilic, electrically neutral polymer, e.g. polyethylene glycol (PEG) being an appropriate choice (Mei et al., 2013; Yu et al., 2017). As for the other requirement, using a star-shaped CA-PLGA/NPs would be highly beneficial.

Ligand-mediated active targeting requires a simple, yet universal linker between the ligands and the NPs in order to flexibly attach versatile ligands to the NPs of interest (Li et al., 2016). However conventional strategies for conjugating ligands to NPs are complex and often inefficient (Park et al., 2014). Two primary processes for the conjugation exist: surface modification of NPs or using pre-functionalized polymers to produce NPs. The former method usually requires coupling agents and reactive linkers, demanding a rigorous purification process to remove excess reactants and catalysts (Narayanan et al., 2010; Takahashi et al., 2010). The latter process is accompanied by time consuming and inefficient alterations of chemical properties, ultimately compromising the efficiency of drug loads (Gullotti and Yeo, 2012). However, a novel, simple and versatile conjugating scheme based on dopamine polymerization may be used for this surface modification to achieve functionalization of NPs with active targeting ligands (Lee et al., 2007). The ligands featuring nucleophilic functional groups (e.g. amine and thiol) could be incorporated into the surface layer of polydopamine (PDA) via Michal addition or Schiff base reactions (Lee et al., 2009; Park et al., 2014). For these reasons, we selected an amine-terminated PEG-FA copolymer as the targeting ligand incorporated into PDA coated CA-PLGA/NPs to ensure efficient passive accumulation (Ibrahim et al., 2017) and simple universal active targeting.

Stimuli responsive drug delivery systems are considered to be promising for the control of drug release *in vivo* (Liu et al., 2017b). Given the pH values variations from physiological pH to slightly acidic pH in normal tissues versus neoplastic tissues (Dai et al., 2011), a pH-triggered strategy is particularly attractive for the design of an anticancer drug delivery system (Luo et al., 2012; Song et al., 2016; Liu et al., 2017a). Su et al. (2011) described a novel pH responsive mechanism based on facile conjugation of the anticancer drug BTZ to catechol containing polymeric carriers. Here, BTZ is bound to catechol under neutral and alkaline conditions and may be released in acid neoplastic tissues or subcellular endosomes. Based on the fact that PDA contains an adequate amount of catechol, we hypothesize that a therapeutic approach combining a positive active targeting strategy with a pH responsive method may result in an improved curative effect.

Docetaxel (DTX) has been approved in the United States by the FDA for the treatment of solid tumors such as breast cancer, ovarian cancer, and cervical cancer. Nevertheless, clinical application of DTX is limited because of its poor aqueous solubility, high toxicity and low bioavailability (Kumari et al., 2010; Wang et al., 2015; Shaw et al., 2017). Bortezomib (BTZ), a clinical anticancer drug, has been used to bind to threonine residues in the active sites of several proteases and generally inhibits the growth of cancer cells. However, the adverse pharmacokinetic effects of BTZ, including nonspecific binding to proteins, rapid clearance from blood, and dose-limiting toxicities, may lead to its limited efficacy against many solid tumors (Su et al., 2011). Therefore, we designed a co-delivery formulation to administer both DTX and BTZ in which the latter was conjugated to DTX-loaded CA-PLGA@PDA-PEG-FA/NPs for the subsequent treatment of breast cancer *in vitro* and *in vivo*. The PDA layer offers a universal mechanism for the conjugation of ligands as well as the construction of a facile pH triggered system. FA, an active targeting ligand for breast cancer, may contribute to overcome some of the main shortcomings of BTZ in solid tumors.

## 2. Materials and methods

### 2.1. Materials

Dopamine hydrochloride, coumarin-6 (C6), IR-780, 3-(4,5-dimethylthiazol-2-yl)-2,5-diphenyltetrazolium bromide (MTT), 2-(4-amidinophenyl)-6-indolecarbamidine dihydrochloride (DAPI) staining solution, acetonitrile and methanol (HPLC grade) were purchased from Sigma-Aldrich (St. Louis, MO, USA). NH_2_-PEG-FA and NH_2_-PEG were purchased from Shanghai Yare Biotech, Inc. (Shanghai, China). Docetaxel (DTX, purity: 99.9%) was purchased from Shanghai Jinhe Bio-Technology Co., Ltd. (Shanghai, China). Bortezomib (BTZ) was purchased from Beijing Zhongshuo Pharmaceutical Technology Development Co., Ltd. (Beijing, China). The Star-shaped copolymer CA-PLGA was synthesized as described previously (Zeng et al., 2013). The human breast carcinoma cell line MCF-7 was purchased from American Type Culture Collection (ATCC, Rockville, MD, USA).

### 2.2. Fabrication of DTX-loaded CA-PLGA/NPs

Docetaxel-loaded CA-PLGA/NPs were prepared via an optimized nanoprecipitation method based on an acetone/water system (Zeng et al., 2013, 2015). First, 100 mg star-shaped polymer CA-PLGA and 10 mg DTX powder were completely dissolved in 8 ml acetone. Then, the solution was added dropwise to 100 ml 0.03% (w/v) TPGS aqueous solution under stirring. The resulting mixture was stirred overnight open to air to evaporate acetone and encapsulate the drugs. Next, the solution was centrifuged at 18,000 rpm for 15 min and washed three times with deionized water to remove non-encapsulated DTX and TPGS emulsifier. Finally, the DTX-loaded CA-PLGA/NPs were lyophilized for further use.

### 2.3. Prime-coating of PDA

The process of PDA coating is carried out in an alkaline environment. Briefly, approximately 50 mg DTX-loaded CA-PLGA/NPs was resuspended in 50 ml Tris buffer (pH 8.5, 10 mM) and 25 mg dopamine hydrochloride was dissolved in 500 μl deionized water. The latter solution was added dropwise to the previous solution under magnetic stirring and the resulting mixture was stirred for 5 h. The solution gradually darkened during the procedure. Then, the mixture was centrifuged at 18,000 rpm for 15 min and washed three times with deionized water to remove uncoated dopamine hydrochloride. Finally, the surface modified NPs were lyophilized and characterized as DTX-loaded CA-PLGA@PDA/NPs.

### 2.4. Conjugation of ligands to PDA-coated NPs

The CA-PLGA@PDA/NPs were resuspened in Tris buffer (pH 8.5, 10 mM) at a concentration of 1 mg/ml. The targeted ligand NH_2_-PEG-FA was added to the solution at a concentration of 2 mg/ml. The mixture was stirred at room temperature for 5 h. Then, the mixture was centrifuged at 18,000 rpm for 15 min and washed three times with deionized water to remove any unreacted ligands. Then, the product was lyophilized and characterized as DTX-loaded CA-PLGA@PDA-PEG-FA/NPs. The CA-PLGA@PDA-PEG/NPs were prepared in the same manner except for replacement of NH_2_-PEG-FA with NH_2_-PEG.

### 2.5. Loading BTZ onto PDA-coated NPs

Briefly, 50 mg of CA-PLGA@PDA-PEG-FA/NPs were resuspened in Tris buffer (pH 8.5, 10 mM) at a concentration of 1 mg/ml and 6 mg BTZ powder was dissolved in 200 μl DMSO. Subsequently, the latter solution was added dropwise to the former solution under stirring. Then, the mixture was stirred overnight and the same centrifugation procedure was repeated as described above. The final product was lyophilized for 48 h and characterized as DTX-loaded CA-PLGA@PDA-PEG-FA + BTZ/NPs.

All fluorescent NPs (C6- or IR-780-loaded NPs) were prepared using a similar procedure except for replacement of DTX with C6 or IR-780.

### 2.6. Characterization of the nanoparticles

The particle size and zeta potential of the NPs were measured by dynamic light scattering (DLS) using a Malvern Mastersizer 2000 (Zetasizer Nano ZS90; Malvern Instruments Ltd., Malvern, UK). After being resuspended in deionized water, the samples were evaluated at room temperature. The surface morphology of the NPs was analyzed by transmission electron microscopy (TEM, Tecnai G2 F30; FEI Company, Hillsboro, OR, USA). A sample solution with appropriate concentration was dropped onto a copper grid coated with a carbon membrane. Then, the copper grid was dried before analysis. The Fourier transform infrared (FT-IR) spectra of drug-free NPs were obtained by FT-IR spectrophotometry (Thermo Nicolet, Madison, WI, USA) using KBr for pelletizing. X-ray photoelectron spectroscopy (XPS) data was collected using a Kratos Axis Ultra DLD spectrometer with monochromatic Al Kα radiation (*hν* = 1486.58 eV). Survey and high-resolution spectra were used for sample analysis. In order to measure the drug loading content (LC), the supernatant of each step was preserved and filtered for subsequent HPLC analysis (LC 1200; Agilent Technologies, Santa Clara, CA, USA). A reverse-phase C-18 column (150 * 4.6 mm, 5 μm, C18; Agilent Technologies, CA, USA) was used for compound separation. The mobile phase consisted of acetonitrile and deionized water (50:50 for DTX, 80:20 for BTZ, V/V). The flow rate of the mobile phase was set to 1.0 ml/min and 20 μl of each sample was transferred to the injector. The UV/Vis detector was set to a detection wavelength of 227 nm and 270 nm for the detection of DTX and BTZ. The LC (%) was calculated using the following equation.

$$\text{LC}(\%)=\frac{\text{Weight }\text{of }\text{DTX/BTZ }\text{in }\text{NPs}}{\text{Weight }\text{of }\text{NPs}}\times 100\mathrm{\%.}$$

### 2.7. *In vitro* drug release profiles

The *in vitro* DTX release from the DTX-loaded NPs was measured using procedures described previously (Zhu et al., 2016). Briefly, 5 mg of lyophilized NPs was resuspended in 1 ml of phosphate buffer solution (PBS, pH = 5.0, 6.5 or 7.4, containing 0.1% w/v Tween 80) to construct an initial release system. Tween 80 was used to increase the solubility of DTX in the buffer. Then, the dispersed solution was transferred into a dialysis membrane bag (MWCO = 3500, Shanghai Sangon, China) that was immersed in 15 ml of PBS release medium (pH = 5.0, 6.5 or 7.4) in a centrifuge tube. Next, the tube was placed in an orbital water bath and shaken at 37 °C. The release medium in the tube was replaced with fresh medium at the designated intervals and the medium containing DTX was filtered for subsequent HPLC analysis. The analysis of *in vitro* BTZ release from DTX-loaded CA-PLGA@PDA-PEG-FA + BTZ/NPs was also performed and the release amount of BTZ was measured by HPLC using the conditions described above. Finally, the accumulative release profiles of DTX and BTZ were plotted against time.

### 2.8. Endocytosis of fluorescent NPs

MCF-7, a breast cancer cell line, was selected to determine the endocytosis behavior of the above NPs. MCF-7 cells were cultured in a dish in Dulbecco’s Modified Eagle’s Medium (DMEM) supplemented with 10% fetal bovine serum (FBS), and incubated in 5% CO_2_ at 37 °C. Coumarin-6 loaded NPs were used to observe and analyze the cellular uptake. Briefly, the cells were incubated with C6-loaded NPs (250 μg/ml) for 0.5 h and 2 h, rinsed three times with PBS and fixed for 15 min with paraformaldehyde (4%, w/v). Then, the nuclei were counterstained with DAPI for 10 min. After being rinsed three times with PBS, the cells were observed by confocal laser scanning microscope (CLSM; Olympus Fluoview FV-1000, Japan) with two channels: a blue channel excited at 340 nm and a green channel excited at 485 nm.

For flow cytometry (FCM), MCF-7 cells were seeded in a 6-well plate at a concentration of 1*10^6^ cells/well. After being incubated with fluorescent NPs (250 μg/ml) for 1 h, the cells were rinsed three times with PBS, digested by trypsin and collected in a tube for FCM (BD Biosciences, San Jose, CA, USA) detection at an excitation wavelength of 488 nm. The fluorescent signal of C6-loaded NPs were recorded at an emission wavelength of 530 nm using approximately 10,000 cells.

### 2.9. Cell viability evaluation

MCF-7 cells were seeded in a 96-well plate at a concentration of 1×10^4^ cells/well. After incubation overnight, the adherence cells were treated with Taxotere^®^, BTZ, DTX + BTZ(1:1), DTX-loaded CA-PLGA@PDA-PEG/NPs, DTX-loaded CA-PLGA@PDA-PEG-FA/NPs, DTX-loaded CA-PLGA@PDA-PEG-FA + BTZ/NPs and BTZ + DTX-loaded CA-PLGA@PDA-PEG-FA/NPs suspension at 0.25, 2.5, 12.5 and 25 μg/ml equivalent DTX concentrations for 24 and 48 h. At the designed time interval, 20 μl of MTT solution (5 mg/ml) was added into each well and the cells were incubated for another 4 h. Then, the media containing MTT was aspired off and 150 μl of DMSO was added into each well to dissolve the formazan crystals. The optical density (OD) value of each well was detected by a microplate reader (Bio-Rad Model 680, UK) at a wavelength of 490 nm. Untreated cells indicated 100% viability and the control group represented zero absorbance. The IC_50_ value is defined as the concentration at which half of the cell population was inhibited under the corresponding conditions. This value was calculated by curve fitting of the OD values compared with 100% viability and zero absorbance.

### 2.10. Pharmacokinetics analysis

The protocols for the conducted animal assays were approved by the Administrative Committee on Animal Research in Tsinghua University. Male Sprague–Dawley (SD) rats of 230 ± 10 g (5–6 weeks old) were adopted for pharmacokinetics analysis and the rats were randomly divided into five groups (*n* = 5) as follows: Taxotere^®^ group, DTX-loaded CA-PLGA@PDA/NPs group, DTX-loaded CA-PLGA@PDA-PEG/NPs group, DTX-loaded CA-PLGA@PDA-PEG-FA/NPs group and DTX-loaded CA-PLGA@PDA-PEG-FA + BTZ/NPs group. At the scheduled intervals, the formulations were intravenously administered to the rats via tail vein injection at an equivalent DTX dose of 10 mg/kg in PBS. 200 μl of blood was collected and centrifuged at 4000 rpm for 10 min. DTX in the plasma samples was extracted into 1 ml of diethyl ether followed by evaporation of the extractant and addition of 100 μl of mobile phase (acetonitrile:deionized water = 50:50, V/V). Subsequently, the mobile phase containing DTX was detected by HPLC as mentioned above. Finally, the curve of blood concentration was plotted against time by reference to the standard curve of DTX. Moreover, a pharmacokinetics analysis of bare BTZ and DTX-loaded CA-PLGA@PDA-PEG-FA + BTZ/NPs was performed.

### 2.11. Animals and tumor model

The protocols for the conducted animal assays have been approved by the Administrative Committee on Animal Research in Tsinghua University. Female, severe combined immunodeficient (SCID) mice, age 3–4 weeks, were purchased from Guangdong Medical Laboratory Animal Center (GDMLAC). After 2–3 weeks of feeding, about 100 μl of MCF-7 cells (2 × 10^6^ cells per mouse) in PBS were implanted subcutaneously into the back area of the mice (16–18 g). Afterwards, the tumor sizes were measured by a vernier caliper and the tumor volumes were calculated using the following formula: 4π/3 × (length/2) × (width/2)^2^.

### 2.12. *In vivo* imaging and biodistribution analysis

For biodistribution analysis, IR-780-loaded CA-PLGA@PDA-PEG/NPs and CA-PLGA@PDA-PEG-FA/NPs were prepared using the methods described above. After the tumor volume increased to 200 mm^3^, the mice were randomly divided into three groups as follows: free IR-780 group, IR-780-loaded CA-PLGA@PDA-PEG/NPs group and IR-780-loaded CA-PLGA@PDA-PEG-FA/NPs group. Each group consisted of three mice. Afterwards, the forementioned three materials were intravenously injected into the mice via tail vein injection at an equivalent IR-780 dose of 1 mg/kg in 100 μl PBS. 4 and 24 h post-injection, biodistribution images were recorded by Maestro™, an automated *in vivo* Imaging System (Cri Maestro™, USA) at an excitation wavelength of 704 nm and an emission wavelength of 740–950 nm. The mice were sacrificed 24 h post-injection and the organs (heart, liver, spleen, lung and kidney) as well as the tumors were collected for further quantification of residual agents.

### 2.13. *In vivo* antitumor effects

After the tumor volume increased to 50 mm^3^, the mice were randomly divided into five groups (*n* = 5) as follows: saline group, Taxotere® group, DTX-loaded CA-PLGA@PDA-PEG/NPs group, DTX-loaded CA-PLGA@PDA-PEG-FA/NPs group and DTX-loaded CA-PLGA@PDA-PEG-FA + BTZ/NPs group. The forementioned five materials were intraperitoneally injected every four days into the mice of each group at a DTX dose of 10 mg/kg in 100 μl PBS. The tumor sizes and weights of the mice were recorded every other day to evaluate the antitumor effect *in vivo*. After 14 days of treatment, the mice were sacrificed and the tumor masses were removed, measured and weighed. The tissues of the organs (heart, liver, spleen, lung and kidney) and the tumors from each group were harvested and fixed in 10% neutral buffered formalin for histology analysis. Finally, the tissues were embedded in paraffin followed by sectioning into about 4 µm slices, staining with hematoxylin and eosin (H&E) and analysis by optical microscopy.

### 2.14. Statistical methodology

All experiments were repeated at least three times unless otherwise noted. The results were expressed as mean ± SD, and the statistical significance of all results was determined by a Student’s *t*-test. The *p* < .05 was considered statistically significant.

## 3. Results and discussion

### 3.1. Preparation of NPs

**Figure 1.**
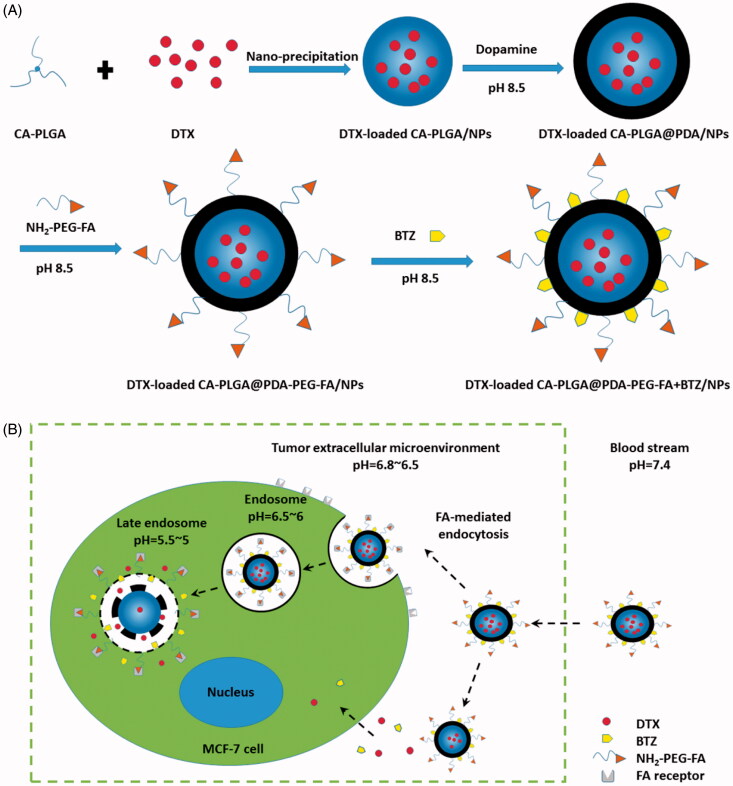
(A) Technological process of the preparation of DTX-loaded CA-PLGA@PDA-PEG-FA + BTZ/NPs; (B) Schematic illustration of FA-mediated endocytosis process and pH triggered intracellular release of DTX-loaded CA-PLGA@PDA-PEG-FA + BTZ/NPs.

As shown in Figure 1, the overall experimental process consisted of DTX loading, PDA coating, ligand conjugation and BTZ loading. DTX loading was based on an optimized and facile nano-precipitation method. According to a report published in 2007 (Lee et al., 2007), a mussel-inspired PDA coating method was applied to numerous surface modifications. Furthermore, as reported in the literatures, the PDA layer may adhere on various substrates, such as gold NPs (Liu et al., 2013b), carbon nanotubes (Zhao et al., 2016), polymer NPs (Zhu et al., 2016) and mesoporous silica NPs (Chang et al., 2016). The PDA coating may also act as a universal bond between the ligands and the NPs in terms of reactivity to amine and thiol groups. In a weakly alkaline solution, the dopamine monomer was oxidized to quinone and polymerized to PDA, ultimately adhering to the surface of NPs (Lee et al., 2007; Postma et al., 2009; Hong et al., 2012). NH_2_-PEG-FA, the targeting ligand used for targeting breast cancer cells and bearing a terminal amine group, could conjugate to PDA coated NPs in a simple reaction. The concept of using a pH triggered strategy in our research was based on promising results obtained from previously published reports (Liu et al., 2013a; Gu et al., 2015). Under alkaline conditions, BTZ could be incorporated into the PDA layer based on the reaction between the boronic acid active site in BTZ and catechol in PDA. This facile conjugation method between BTZ and polymers with catechol may inhibit the activity of BTZ and therefore reduce nonspecific cell endocytosis of the drug. Moreover, considering structural characteristics, BTZ may be released under acidic conditions (cf. Supplementary Information, Figure S1) and may also promote drug release at the tumor site with a selective increase in antitumor activity. In a word, the targeting material, DTX-loaded CA-PLGA@PDA-PEG-FA + BTZ/NPs, contains several beneficial properties for antitumor therapy, including active targeting, pH responsiveness, prolonged *in vivo* circulation and dual drug loading capacity.

### 3.2. Characteristics of NPs

**Figure 2.**
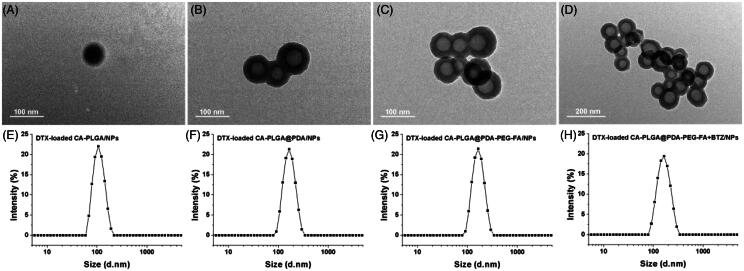
TEM images of (A) DTX-loaded CA-PLGA/NPs, (B) DTX-loaded CA-PLGA@PDA/NPs, (C) DTX-loaded CA-PLGA@PDA-PEG-FA/NPs and (D) DTX-loaded CA-PLGA@PDA-PEG-FA + BTZ/NPs. The size distribution by intensity of (E) DTX-loaded CA-PLGA/NPs, (F) DTX-loaded CA-PLGA@PDA/NPs, (G) DTX-loaded CA-PLGA@PDA-PEG-FA/NPs and (H) DTX-loaded CA-PLGA@PDA-PEG-FA + BTZ/NPs.

The size and surface zeta potential of the NPs, both parameters playing an important role in drug release, cellular uptake, pharmacokinetics and biodistribution, were measured by dynamic light scattering (DLS) as highlighted in Table 1. The hydrodynamic sizes were found to be slightly increased due to the PDA coating, ligand conjugation and BTZ loading. This phenomenon indicates that PDA, ligands and BTZ were successfully incorporated. Moreover the proper size and narrow size distribution (PDI <0.15) may promote an accumulation of the NPs in the tumors via EPR effect (Torchilin, 2011). Some of the size distributions of the NPs are shown in Figure 2, others are presented in Figure S2 (cf. Supplementary Information).

**Table 1.** Characterization of DTX-loaded NPs.

Samples (*n* = 3)	Size(d nm)	PDI	ZP (mV)	DTX LC (%)	BTZ LC (%)
CA-PLGA/NPs	103.4 ± 2.8	0.115	−22.50 ± 4.3	9.96 ± 0.34	N.A.
CA-PLGA@PDA/NPs	160.8 ± 3.4	0.122	−18.40 ± 4.1	9.78 ± 0.28	N.A.
CA-PLGA@PDA-PEG/NPs	164.5 ± 3.1	0.119	−12.40 ± 3.2	9.65 ± 0.32	N.A.
CA-PLGA@PDA-PEG-FA/NPs	166.4 ± 3.9	0.139	−11.70 ± 3.8	9.67 ± 0.45	N.A.
CA-PLGA@PDA-PEG-FA + BTZ/NPs	168.7 ± 4.2	0.146	−11.20 ± 3.6	9.01 ± 0.25	9.18 ± 0.41

PDI: polydispersity index; ZP: zeta potential; LC: loading content; NA: not applicable, *n* = 3.

The zeta potential value represents a critical parameter for measuring the stability of NPs in solution. As shown in Table 1, the zeta potential of DTX-loaded CA-PLGA/NPs and DTX-loaded CA-PLGA@PDA/NPs were −22.5 and −18.4 mV, respectively. These values are slightly lower than that of DTX-loaded CA-PLGA@PDA-PEG/NPs, DTX-loaded CA-PLGA@PDA-PEG-FA/NPs and DTX-loaded CA-PLGA@PDA-PEG-FA + BTZ/NPs (approximately -12.0 mV). We believe that the carboxylate function in CA-PLGA and the deprotonation of the phenolic hydroxyl groups on the PDA layer may have contributed to the negative potential. In addition, the polymer segments PEG decreased the absolute value of the zeta potential because the PEG could shield partial negative charge, and the zeta potential of CA-PLGA@PDA-PEG-FA/NPs has no significant change after conjugating BTZ. Overall, the negative value for the zeta potential resulted in a reduction in clearance of the NPs by the reticuloendothelial system (RES) and further increased the half-life of *in vivo* circulation as well as the tumor accumulation.

The loading content (LC) represents an important indicator for the measurement of the drug carrying capacity of NPs. As shown in Table 1, the surface modification did not significantly reduce the LC. We found that all NPs were able to load approximately 10% of DTX. As for BTZ, the DTX-loaded CA-PLGA@PDA-PEG-FA/NPs could load approximately 9% of BTZ upon method optimization.

TEM was adopted to observe the morphology of the NPs. The material exhibited a nearly spherical shape with smooth surface. Figure 2(A–D) show the TEM pictures of DTX-loaded CA-PLGA/NPs, DTX-loaded CA-PLGA@PDA/NPs, DTX-loaded CA-PLGA@PDA-PEG-FA/NPs and DTX-loaded CA-PLGA@PDA-PEG-FA + BTZ/NPs, respectively. The image of the DTX-loaded CA-PLGA@PDA/NPs exhibited a core-shell structure and a uniform size, indicating that any auto-polymerization reactions for dopamine was negligible and the PDA layer successfully adhered to the NPs. In addition, the size values as measured by TEM were smaller than the hydrodynamic size values obtained by DLS. According to previously published literature (Tao et al., 2014), this finding may due to shrinkage and collapse upon drying of the NPs.

For controlling loading of DTX and BTZ to NPs at the ratio, the pre-experiment was introduced to determine the compatible reaction condition. As shown in Figure S3 (cf. Supplementary Information), the final reaction concentration of BTZ was 6 mg/50 ml.

### 3.3. FT-IR and XPS analysis

**Figure 3.**
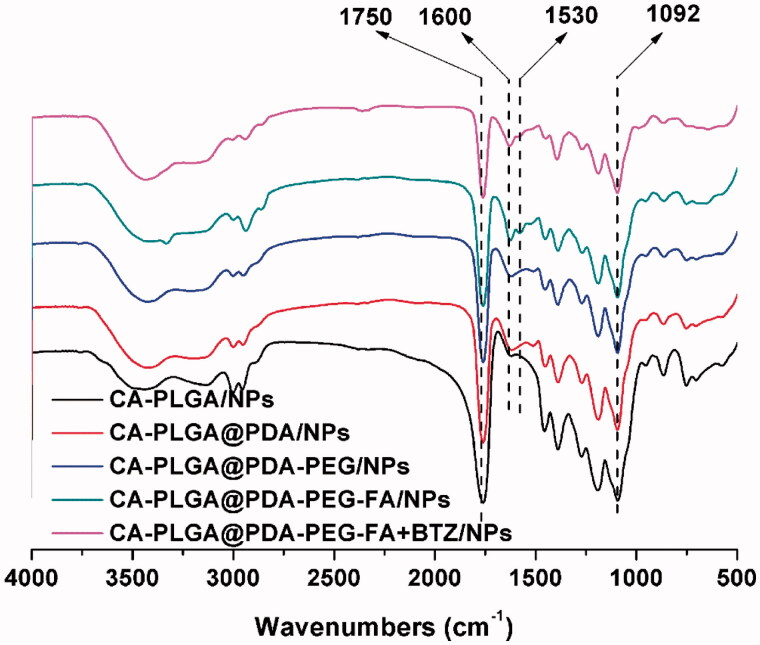
FT-IR spectra of all NPs.

FT-IR spectroscopy was adopted as a method to confirm PDA coating and ligand conjugation. As shown in Figure 3, the intense signal at 1750 cm^−1^ that appeared in all spectra may be ascribed to the presence of a carbonyl group in CA-PLGA. Several other absorption peaks appeared upon surface modification. The peaks at 1600 cm^−1^ and 1530 cm^−1^ could be attributed to the overlap of the C = C resonance vibrations in the aromatic rings and the N-H bending vibrations, providing further evidence for a successful coating with PDA (Liu et al., 2016). The broad signal between 3600 cm^−1^ to 3300 cm^−1^ was caused by the stretching vibrations of N-H/O-H. The peak at 1092 cm^−1^ corresponded to the stretching vibrations of C-O-C. The latter two peaks confirmed a successful modification with NH_2_-PEG or NH_2_-PEG-FA.

**Figure 4.**
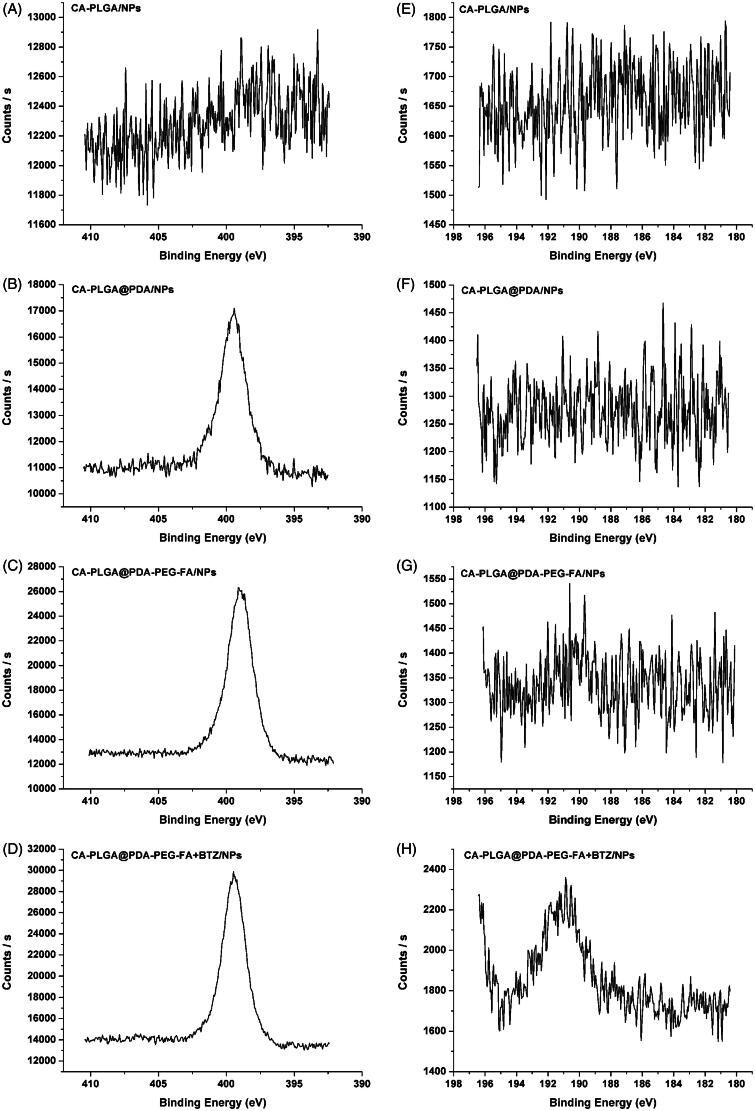
XPS spectra of NPs. (A–D) narrow scan of N1s peaks; (E–H) narrow scan of B1s peaks.

X-ray photoelectron spectroscopy was used to further confirm the surface modification of the NPs upon BTZ loading. As shown in Figure 4(A–D), the observed nitrogen peak (N1s) at 399.49 eV (except for CA-PLGA/NPs), provided more evidence for a successful PDA coating reaction. For the carbon atom (C1s), three peaks were observed as shown in Figure S4 (cf. Supplementary Information), including the signal for O = C–O at 288.8 eV, the signal for C–O/C–N at 286.2 eV, and the signal for C–C at 284.8 eV. Accompanied by PDA coating and ligand conjugation, the signal intensity at 288.8 eV was weaker and the peaks at 286.2 and 284.8 eV overlapped. This phenomenon indicated an increasing proportion of C–O/C–N and C–C, as well as a decreasing proportion of O = C–O. According to the data shown in Figure 4(E–H), the boron peak (B1s) at 190.88 eV could only be found in the spectrum of CA-PLGA@PDA-PEG-FA + BTZ/NPs, demonstrating a successful BTZ loading. The survey spectra of all NPs can be found in Figure S4 (cf. Supplementary Information).

### 3.4. *In vitro* drug release

The drug release behavior affects the effectiveness and controllability of a drug delivery system as well as the method of administration. Considering varying pH values at different physiological circumstances and the pH sensitivity of the catechol-boronate bond, we designed a series of release media with different pH values for simulation of the various release sites *in vivo*. Examples include pH 7.4 for blood stream, pH 6.5 for tumor extracellular microenvironment and pH 5.0 for late endosomes or lysosomes (Zhou et al., 2011).

**Figure 5.**
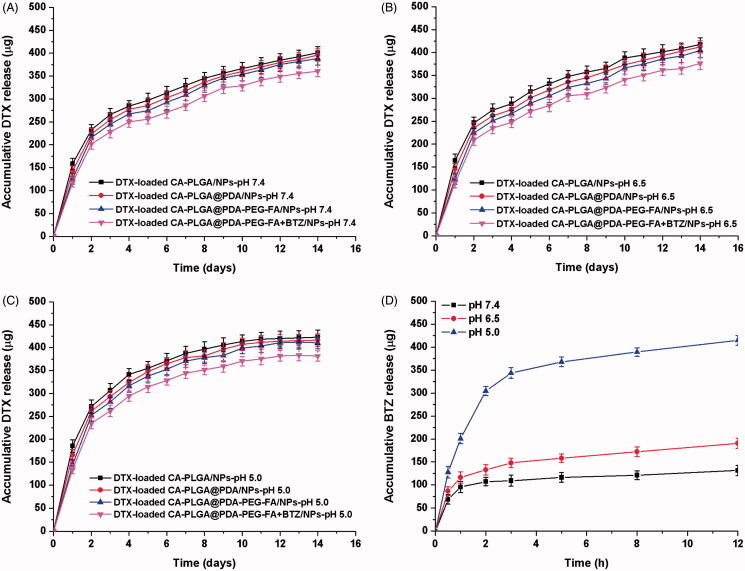
In vitro DTX release profile of DTX-loaded CA-PLGA/NPs, DTX-loaded CA-PLGA@PDA/NPs, DTX-loaded CA-PLGA@PDA-PEG-FA/NPs, and DTX-loaded CA-PLGA@PDA-PEG-FA + BTZ/NPs in release medium with different pH values: (A) pH 7.4; (B) pH 6.5; (C) pH 5.0. (D) *In vitro* BTZ release profile of DTX-loaded CA-PLGA@PDA-PEG-FA + BTZ/NPs in release medium with different pH values.

As shown in Figure 5(A–C), the release profiles of DTX from NPs exhibited a similar release tendency at all screened pH values and the drug release profile could be separated into two individual stages. The first phase lasted approximately two days and almost lead to an overall DTX release of 52%. Then, the release rate decreased and nearly 80% of all DTX accumulated in the media after two weeks. All release curves, regardless of sample type or pH value, indicated a similar release pattern for DTX and a similar final release amount. The only noticeable difference was that the release rates of PDA coated NPs was found to be increased and approached a level similar to that of DTX-loaded CA-PLGA/NPs upon lowering the pH value from 7.4 to 5.0. Presumably, this finding was caused by the removal of the PDA coating under acidic conditions. The displayed release pattern of DTX from the drug carrier vehicle may satisfy clinical requirements.

Figure 5(D) showed the release behavior of BTZ from DTX-loaded CA-PLGA@PDA-PEG-FA + BTZ/NPs at different pH conditions. At neutral pH, BTZ exhibited a very slow release rate and only about 25% BTZ was released after 12 h. Upon lowering the pH value to 6.5, the release rate and accumulative release rate exhibited a slight increase. Upon lowering the pH value to 5.0, the release rate of BTZ accelerated significantly and more than 50% of BTZ accumulated in the release media within 2 h. Approximately 80% of BTZ was found to be released from the DTX-loaded CA-PLGA@PDA-PEG-FA + BTZ/NPs after 12 h. This release behavior of BTZ highlights the pH sensitivity of the catechol-BTZ bond contributing to the accumulation of BTZ at the tumor sites, potentially resulting in an improved treatment effect.

In order to mimic the condition from blood vessels to tumor sites or endosomes, we invested the release of BTZ upon shifting of pHs from neutral to acidic condition and the result was presented in Figure S5 (cf. Supplementary Information). Although there was little BTZ accumulative release after 4 hours under pH 7.4, more than 100 μg of BTZ were released when the pH value was shifted to 6.5. More obviously there was a burst release of BTZ upon the shifting of pH from 6.5 to 5.0. The release pattern was conductive to reduce the BTZ leakage during the circulation in blood and increase BTZ enrichment in tumor sites or endosomes for better tumor killing effect.

### 3.5. Cellular uptake of NPs

**Figure 6.**
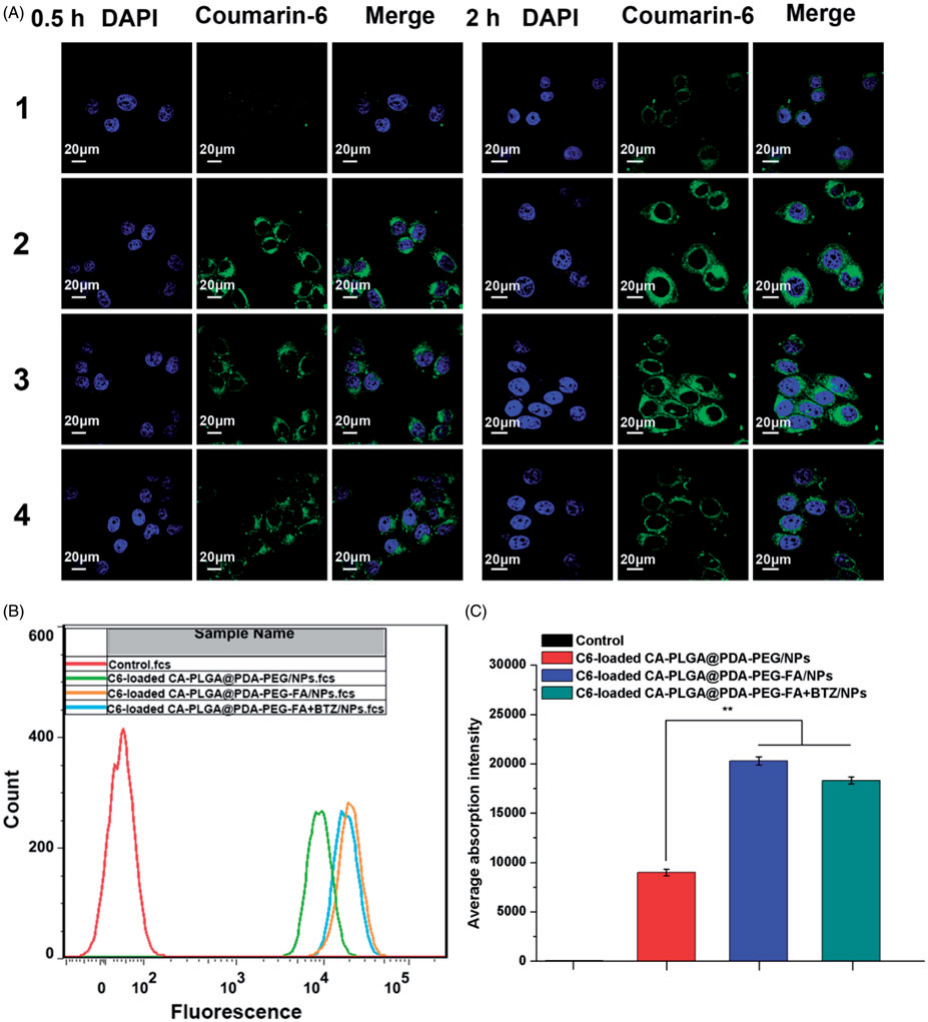
Cellular uptake of coumarin-6 loaded NPs. (A) CLSM images for MCF-7 cells after 0.5 h-incubation and 2 h-incubation. 1–4 represent coumarin-6 loaded CA-PLGA@PDA-PEG/NPs, CA-PLGA@PDA-PEG-FA/NPs, CA-PLGA@PDA-PEG-FA + BTZ/NPs and coumarin-6 loaded CA-PLGA@PDA-PEG-FA + BTZ/NPs with free FA. Scale bar: 20 μm. (B) Graphic demonstration of FCM analysis of MCF-7 cells after 1 h-incubation. (C) Quantitative FCM histogram (*t*-test, **p < .01).

The cellular internalization performance and sustained retention behavior were investigated by both qualitative and quantitative measures. DTX in the composition was replaced with a fluorescent probe, namely coumarin-6 (C6). The dye may be observed by Confocal Laser Scanning Microscopy (CLSM) and detected by flow cytometry (FCM). Figure 6(A) shows the CLSM images of MCF-7 cells treated with fluorescent NPs (250 μg/ml, resuspended in DMEM) for 0.5 h and 2 h. It is believed that NPs entered the cells and distributed in the periphery of the nucleus, with the blue section representing the nucleus stained by DAPI and the green section indicating coumarin-6 on behalf of the NPs. After 0.5 h of incubation, the fluorescence intensity of C6-loaded CA-PLGA@PDA-PEG/NPs was found to be significantly lower than that of C6-loaded CA-PLGA@PDA-PEG-FA/NPs. With a culture time of up to two hours, the gap of fluorescence intensity was further widened. This phenomenon is believed to be caused by the interactions of the targeting group (FA) and the folate receptors (FRs) on the surface of MCF-7 cells. In order to validate this hypothesis, a competition assay was carried out. Here, the C6-loaded CA-PLGA@PDA-PEG-FA/NPs (125 μg/ml) and free folate (125 μg/ml) were mixed in DMEM. The fluorescence intensity was found to be significantly decreased compared to C6-loaded CA-PLGA@PDA-PEG-FA/NPs, further providing evidence for the hypothesis that the active targeting process was indeed efficient. Furthermore, we noticed that the fluorescence intensity of C6-loaded CA-PLGA@PDA-PEG-FA + BTZ/NPs remained consistent compared to C6-loaded CA-PLGA@PDA-PEG-FA/NPs, indicating that BTZ loading did not significantly influence the interaction between FA and FRs.

To quantitatively validate the above described phenomenon, MCF-7 cells were treated with fluorescent NPs for 1 h and gathered for FCM analysis. As shown in Figure 6(B,C), the fluorescence intensity of the active targeting groups were numerically higher than the non-targeting groups. Once again, the results demonstrated that the active targeting moiety was crucial and the BTZ loaded NPs exhibited similar targeting properties as the active targeting NPs.

### 3.6. Effect of NPs on cell viability

**Figure 7.**
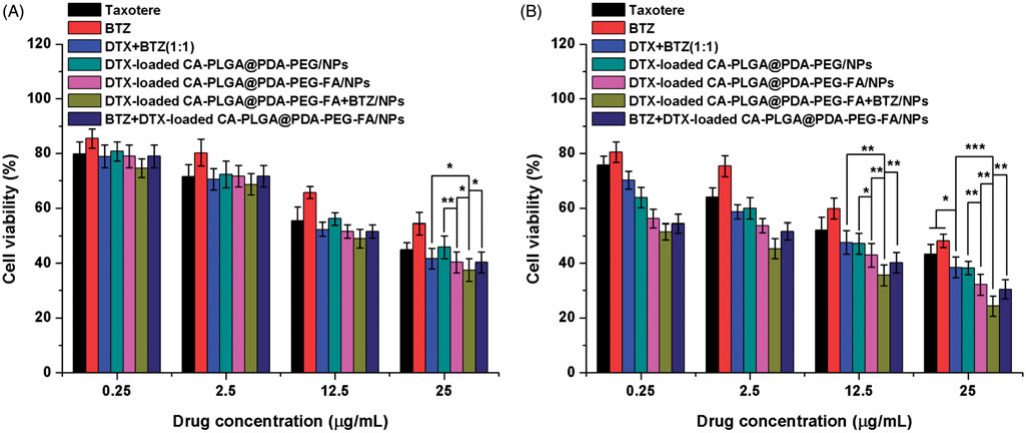
Viability of MCF-7 cells cultured with DTX-loaded NPs in comparison with Taxotere^®^, BTZ, and DTX + BTZ(1:1) at the same dose for (A) 24 h and (B) 48 h (*t*-test, *p < .05, **p < .01, ***p < .001).

In order to evaluate the cytotoxicity of DTX-loaded CA-PLGA@PDA-PEG/NPs, DTX-loaded CA-PLGA@PDA-PEG-FA/NPs, DTX-loaded CA-PLGA@PDA-PEG-FA + BTZ/NPs, and BTZ + DTX-loaded CA-PLGA@PDA-PEG-FA/NPs *in vitro*, a MTT assay was carried out using MCF-7 cells. Drug-free CA-PLGA@PDA-PEG-FA/NPs was investigated to eliminate potential toxicity characteristics of the drug vehicle. As shown in Figure S6 (cf. Supplementary Information), the drug-free CA-PLGA@PDA-PEG-FA/NPs exhibited a survival rate of more than 90% at all concentrations and time periods, eliminating the effects of the vehicle on cell viability. In addition, Taxotere^®^, a clinical DTX formulation, and bortezomib (BTZ) were introduced as references. MCF-7 cells were treated with Taxotere^®^, BTZ, DTX + BTZ (1:1) and the nanoformulations using a drug concentration series of 0.25, 2.5, 12.5, and 25 μg/ml and time preriods of 24 h (cf. Figure 7(A)) and 48 h (cf. Figure 7(B)). The following results were obtained: (1) the drug-loaded NPs, BTZ, DTX + BTZ (1:1) and Taxotere^®^ exhibited cytotoxicity that was found to be dependent on the dose (from 0.25 to 25 μg/ml) and time (24 h and 48 h); (2) after 24 h or 48 h of incubation, it is evident that the survival rate of the active targeting group, DTX-loaded CA-PLGA@PDA-PEG-FA/NPs, was lower than that of non-targeted group, DTX-loaded CA-PLGA@PDA-PEG/NPs. This phenomenon demonstrated that the FA-mediated active targeting mechanism contributed to kill more MCF-7 cells relative to the passive accumulation process alone; (3) after 48 h of incubation, compared to the administration of DTX (alone) or BTZ (alone), the direct co-administration of DTX and BTZ increased the inhibition rate, indicating stronger cytotoxicity; (4) after 24 h and 48 h of incubation, the group that treated with BTZ and DTX-loaded CA-PLGA@PDA-PEG-FA/NPs exhibited the similar inhibitory rate with the group that treated with DTX-loaded CA-PLGA@PDA-PEG-FA/NPs, which was lower than the dual drug-loaded NPs. The poor cytotoxicity may due to the nonspecific binding of BTZ to proteins, (5) after 48 h of incubation, it is significant that the active targeting formulation with dual drug loading, DTX-loaded CA-PLGA@PDA-PEG-FA + BTZ/NPs, exhibited the lowest survival rate among all the selected nanoformulations and clinical drug, demonstrating the highest cytotoxity.

Besides the histogram shown above, the experimental data could be transformed into quantitative IC_50_ values for comparing the effects, which is defined as the drug inhibitor concentration contributing 50% of tumor cell mortality in a certain time period. The calculated IC_50_ values at 24 h and 48 h are listed in Table 2 and are consistent with the results obtained from previous analysis. Overall, DTX-loaded CA-PLGA-PDA-PEG-FA + BTZ/NPs exhibited a great potential for the future treatment of MCF-7 breast cancer.

**Table 2.** IC_50_ values of Taxotere^®^ and DTX-loaded NPs on MCF-7 cells after 24 h- and 48 h-incubation.

	IC50 (μg/ml)			
Incubation time (h)	Taxotere^®^	CA-PLGA@PDA-PEG/NPs	CA-PLGA@PDA-PEG-FA/NPs	CA-PLGA@PDA-PEG-FA + BTZ/NPs
24	20.77 ± 1.84	22.91 ± 2.16	13.79 ± 1.12	9.83 ± 0.51
48	13.57 ± 1.05	5.77 ± 0.37	1.69 ± 0.09	0.52 ± 0.04

### 3.7. *In vivo* imaging and biodistribution of NPs

An *in vivo* imaging system based on near-infrared (NIR) was adopted to capture whole animal images and separated organs at predetermined intervals. For near-infrared imaging and observation, IR-780, a stable near-infrared fluorescent probe, was adopted and the IR-780 loaded NPs were prepared using a similar process as described for DTX-loaded NPs. Free IR-780, IR-780-loaded CA-PLGA@PDA-PEG/NPs and IR-780-loaded CA-PLGA@PDA-PEG-FA/NPs were intravenously administered into the mice via tail vein injection.

**Figure 8.**
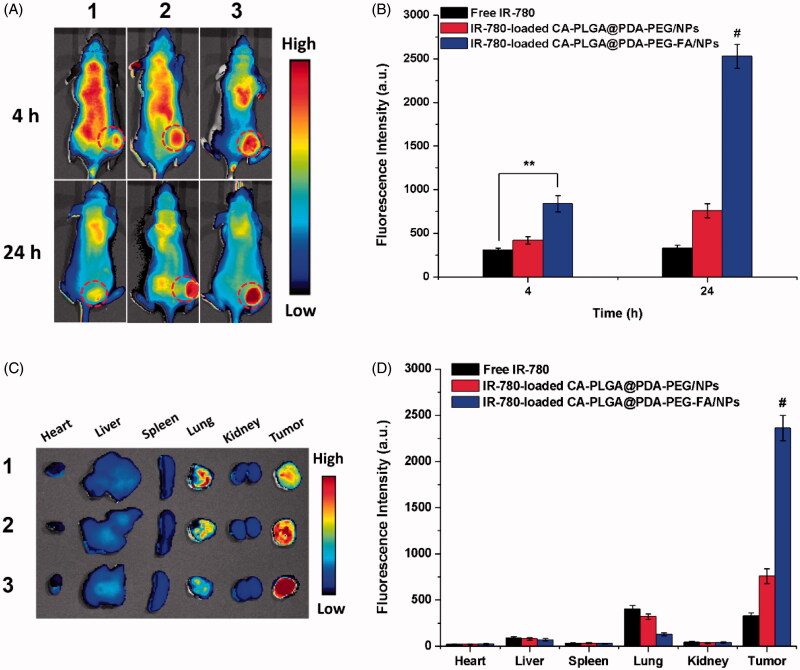
*In vivo* imaging and biodistribution analysis of nude mice bearing MCF-7 cells xenograft after tail vein injection of (1) free IR-780, (2) IR-780-loaded CA-PLGA@PDA-PEG/NPs and (3) CA-PLGA@PDA-PEG-FA/NPs. (A) Time-lapse NIR fluorescence images of nude mice. The tumors were cycled with dotted line. (B) NIR fluorescence intensity of tumors was quantified at indicated time points. (C) NIR fluorescence images of major organs and tumors at 24 h post-injection. (D) Semiquantitative biodistribution analysis in nude mice (*t*-test, **p < .01 between each other, #*p* < . 01 as compared with all others).

The images of whole nude mice with a MCF-7 cell xenograft tumor model are shown in Figure 8(A) and the fluorescence intensity of the tumor sites are shown in Figure 8(B). 4 h post-injection, the fluorescence was recorded throughout the body in all groups. Simultaneously, free IR-780 and the NPs were found to be more abundant at the tumor sites (red circles). Due to the proper size of NPs and the active targeting function of conjugated FA, the fluorescence intensity at the tumor sites of the NPs was stronger than that of free IR-780. Particularly IR-780-loaded CA-PLGA@PDA-PEG-FA/NPs exhibited the strongest fluorescence at the tumor sites. 24 h post-injection, a weaker fluorescence signal throughout the body and a stronger fluorescence signal at the tumor sites could be recorded in the NPs groups. Due to the surface modification with hydrophilic PEG, less NPs were found to be cleared during circulation and more NPs were found to be concentrated at the tumor sites compared to free IR-780. Presumably, the IR-780-loaded CA-PLGA@PDA-PEG-FA/NPs demonstrated the best active targeting performance and tumor site accumulation.

After imaging 24 h post-injection, the animals were sacrificed and the main organs as well as tumors were separated and analyzed by NIR imaging. As shown in Figure 8(C,D), the residual fluorescence in the tumors further demonstrated the highest accumulation of IR-780-loaded CA-PLGA@PDA-PEG-FA/NPs compared to free IR-780 and IR-780-loaded CA-PLGA@PDA-PEG/NPs. Furthermore, residual material was observed in the lungs, potentially caused by mechanical lung capillary retention as free IR-780 and NPs passed through the lungs upon blood circulation.

### 3.8. Pharmacokinetic analysis and *in vivo* anticancer effect of NPs

A pharmacokinetics analysis was adopted to study the valid amount and circulation time of DTX after *in vivo* administration. As shown in Figure S7 (cf. Supplementary Information), using the same DTX dose, the DTX-loaded NPs featured a larger DTX concentration and prolonged blood circulation compared to Taxotere^®^. In particular, an improved blood circulation performance was demonstrated by the NPs modified with PEG. The latter finding is most notably due to the enhanced stability and stealth effects expressed by this material. In addition, the pharmacokinetics behavior of BTZ was studied *in vivo* and the corresponding profiles are shown in Figure S8 (cf. Supplementary Information). Presumably, BTZ conjugated to NPs (DTX-loaded CA-PLGA@PDA-PEG-FA + BTZ/NPs), leading to a prolonged blood circulation time compared to unconjugated BTZ. The active targeting nanoplatform, encapsulating DTX and BTZ, enabled the drugs to accumulate at the tumor sites even after the longer blood circulation times *in vivo*.

**Figure 9.**
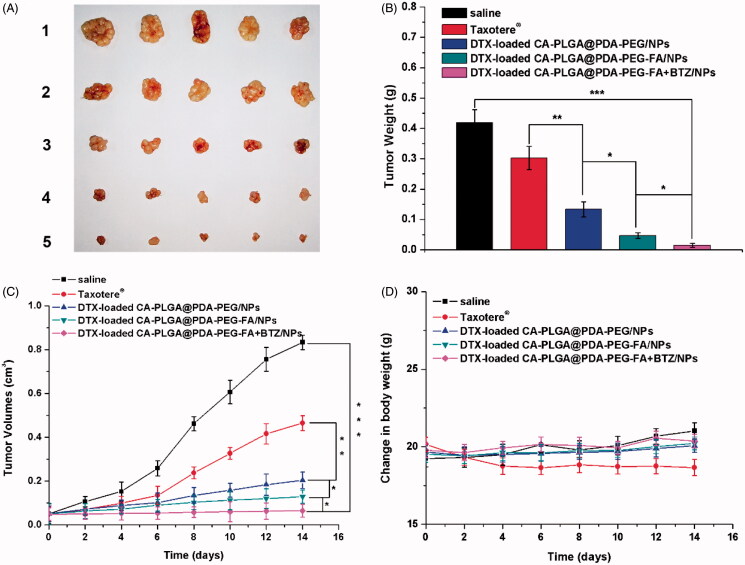
*In vivo* antitumor effect of drug-loaded NPs and Taxotere^®^ on the SCID mice bearing MCF-7 cells xenograft at the experimental period (*n* = 5). (A) Morphology of tumors separated from the executed mice at the end of medication cycle. 1–5 represent saline, Taxotere^®^, DTX-loaded CA-PLGA@PDA-PEG/NPs, CA-PLGA@PDA-PEG-FA/NPs and CA-PLGA@PDA-PEG-FA + BTZ/NPs. (B) Tumors weight of each group at the end of medication cycle (*t*-test, *p < .05, **p < .01, ***p < .001). (C) Tumor growth curve after injected with drug-loaded NPs and Taxotere^®^ (*t*-test, *p < .05, **p < .01, ***p < .001). (D) Change in body weight during the medication cycle.

The following evaluation of the antitumor effect *in vivo* was conducted to further confirm the cytotoxicity results carried out *in vitro*. After 14 days of treatment with the predefined NP formulations, the mice were sacrificed and the tumors were separated and weighed. As shown in Figure 9(A,B), all tumors from the groups treated with the DTX formulations and Taxotere^®^ were smaller and lighter than those from the group treated with saline as control. The following conclusions could be drawn from the data as shown in Figure 9(A–C): (1) the tumor inhibitory effects of the drug-loaded NPs were found to be significantly improved over Taxotere^®^, indicating a higher efficacy; (2) the tumors exhibited smaller sizes upon treatment with DTX-loaded CA-PLGA@PDA-PEG-FA/NPs due to the binding interactions between the ligand FA and FRs overexpressed in MCF-7 cells; (3) after treatment with DTX-loaded CA-PLGA@PDA-PEG-FA + BTZ/NPs, which co-delivering with DTX and BTZ, the tumors did not significantly grow, and the overall size remained constant throughout the treatment cycle. Moreover, the administration of drug-loaded NPs exhibited very little effects on the body weight of the animals compared to the control group treated with saline. However, the use of Taxotere^®^ resulted in a reduction of body weight upon administration (cf. Figure 9(D)).

**Figure 10.**
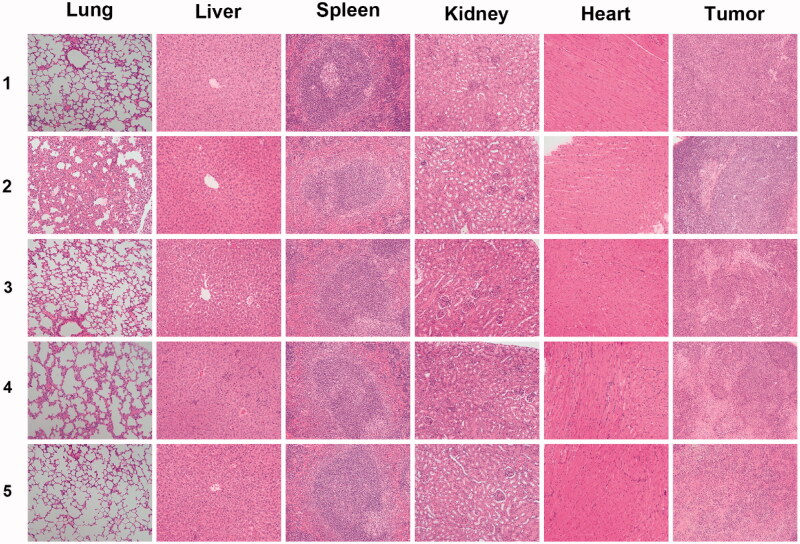
Representative H&E stained images of major organs and tumors after treated with (1) saline, (2) Taxotere^®^, (3) DTX-loaded CA-PLGA@PDA-PEG/NPs, (4) DTX-loaded CA-PLGA@PDA-PEG-FA/NPs and (5) DTX-loaded CA-PLGA@PDA-PEG-FA + BTZ/NPs for 14 days.

In order to further investigate the organ effect of the formulations, histological sections of different organs, including heart, liver, spleen, lung and kidney, as well as tumors were analyzed. As shown in Figure 10, no significant tissue damages could be observed in the sections of the control group treated with saline. As for the Taxotere^®^ group, slight damages could be observed in the lung and liver sections, indicating that this clinically used drug inhibited the tumor growth but associated with side effects on normal organs. The analysis and comparison of the drug-loaded NPs revealed that almost no significant damages could be observed in normal organ tissues, while large-area apoptotic and necrotic regions could be observed in the analyzed tumor sections.

Based on the above analysis, the antitumor effect sequence *in vivo* remained consistent with the carried out cytotoxicity assays. The targeted formulation DTX-loaded CA-PLGA@PDA-PEG-FA + BTZ/NPs almost completely inhibited tumor growth and exhibited almost no notable toxicity on main organs as judged by histological analysis.

## 4. Conclusions

The targeting nanoformulation used in this study (DTX-loaded CA-PLGA@PDA-PEG-FA + BTZ/NPs) was synthesized via the following steps: DTX loading, PDA coating, ligand conjugation and BTZ loading. All NPs exhibited a similar drug LC (%) and drug release profile as well as negative zeta potential. After PDA coating and ligand conjugation, the NPs featured a larger overall size compared to CA-PLGA/NPs. The proper size and NH_2_-PEG-FA ligands could separately promote passive accumulation and active targeting which was demonstrated by a cellular uptake assay *in vitro* and biodistribution studies *in vivo*. Moreover, the binding ability of BTZ exhibited not only a negligible impact on cellular uptake but also a significant increase in cancer-killing rates both *in vitro* and *in vivo*. Furthermore, the properties of the target formulation may enhance the biological utilization of drugs and, potentially, reduce side effects of chemotherapeutic drugs. The list of properties include: long-time *in vivo* circulation (PEG), active targeting (FA), pH responsiveness (BTZ-PDA) as well as dual drug loading and release capacity (DTX & BTZ). In conclusion, this novel nanoplatform which decorated with PDA offers a great potential for the treatment of breast cancer and the overall strategy may also be applicable in various other applications of NPs.

## Supplementary Material

IDRD_Zeng_et_al_Supplemental_Content.docx

## References

[CIT0001] Au KM, Satterlee A, Min Y, et al. (2016). Folate-targeted pH-responsive calcium zoledronate nanoscale metal-organic frameworks: Turning a bone antiresorptive agent into an anticancer therapeutic. Biomaterials 82:178–93. 26763733 10.1016/j.biomaterials.2015.12.018 PMC4728024

[CIT0002] Bertrand N, Wu J, Xu X, et al. (2014). Cancer nanotechnology: the impact of passive and active targeting in the era of modern cancer biology. Adv Drug Deliv Rev 66:2–25. 24270007 10.1016/j.addr.2013.11.009 PMC4219254

[CIT0003] Chang D, Gao Y, Wang L, et al. (2016). Polydopamine-based surface modification of mesoporous silica nanoparticles as pH-sensitive drug delivery vehicles for cancer therapy. J Colloid Interface Sci 463:279–87. 26550786 10.1016/j.jcis.2015.11.001

[CIT0004] Dai J, Lin S, Cheng D, et al. (2011). Interlayer-crosslinked micelle with partially hydrated core showing reduction and pH dual sensitivity for pinpointed intracellular drug release. Angew Chem Int Ed Engl 50:9404–8. 21898731 10.1002/anie.201103806

[CIT0005] Feng L, Liu L, Lv F, et al. (2014). Preparation and biofunctionalization of multicolor conjugated polymer nanoparticles for imaging and detection of tumor cells. Adv Mater 26:3926–30. 24643872 10.1002/adma.201305206

[CIT0006] Geszke M, Murias M, Balan L, et al. (2011). Folic acid-conjugated core/shell ZnS:Mn/ZnS quantum dots as targeted probes for two photon fluorescence imaging of cancer cells. Acta Biomater 7:1327–38. 20965282 10.1016/j.actbio.2010.10.012

[CIT0007] Gu X, Zhang Y, Sun H, et al. (2015). Mussel-inspired polydopamine coated iron oxide nanoparticles for biomedical application. J Nanomater 2015:3.

[CIT0008] Gullotti E, Yeo Y. (2012). Beyond the imaging: limitations of cellular uptake study in the evaluation of nanoparticles. J Control Release 164:170–6. 22568932 10.1016/j.jconrel.2012.04.042 PMC3447109

[CIT0009] He S, Cen B, Liao L, et al. (2017). A tumor-targeting cRGD-EGFR siRNA conjugate and its anti-tumor effect on glioblastoma in vitro and in vivo. Drug Deliv 24:471–81. 28181832 10.1080/10717544.2016.1267821 PMC8241002

[CIT0010] Hong S, Na YS, Choi S, et al. (2012). Non-covalent self-assembly and covalent polymerization co-contribute to polydopamine formation. Adv Funct Mater 22:4711–7.

[CIT0011] Ibrahim SS, Osman R, Mortada ND, et al. (2017). Passive targeting and lung tolerability of enoxaparin microspheres for a sustained antithrombotic activity in rats. Drug Deliv 24:243–51. 28156170 10.1080/10717544.2016.1245368 PMC8241188

[CIT0012] Kamaly N, Xiao Z, Valencia PM, et al. (2012). Targeted polymeric therapeutic nanoparticles: design, development and clinical translation. Chem Soc Rev 41:2971–3010. 22388185 10.1039/c2cs15344k PMC3684255

[CIT0013] Kumari A, Yadav SK, Yadav SC. (2010). Biodegradable polymeric nanoparticles based drug delivery systems. Coll Surf B-Biointerfaces 75:1–18. 10.1016/j.colsurfb.2009.09.001 19782542

[CIT0014] Lee H, Dellatore SM, Miller WM, Messersmith PB. (2007). Mussel-inspired surface chemistry for multifunctional coatings. Science 318:426–30. 17947576 10.1126/science.1147241 PMC2601629

[CIT0015] Lee H, Rho J, Messersmith PB. (2009). Facile conjugation of biomolecules onto surfaces via mussel adhesive protein inspired coatings. Adv Mater Weinheim 21:431–4. 10.1002/adma.200801222 PMC2755254 19802352

[CIT0016] Li J, Wang FS, Sun DQ, Wang RM. (2016). A review of the ligands and related targeting strategies for active targeting of paclitaxel to tumours. J Drug Target 24:590–602. 26878228 10.3109/1061186X.2016.1154561

[CIT0017] Liu G, Tsai HI, Zeng XW, et al. (2017a). Phosphorylcholine-based stealthy nanocapsules enabling tumor microenvironment-responsive doxorubicin release for tumor suppression. Theranostics 7:1192–203. 28435458 10.7150/thno.17881 PMC5399586

[CIT0018] Liu L, Yao W, Rao Y, et al. (2017b). pH-Responsive carriers for oral drug delivery: challenges and opportunities of current platforms. Drug Deliv 24:569–81. 28195032 10.1080/10717544.2017.1279238 PMC8241197

[CIT0019] Liu Y, Wu X, Mi YS, et al. (2017c). PLGA nanoparticles for the oral delivery of nuciferine: preparation, physicochemical characterization and in vitro/in vivo studies. Drug Deliv 24:443–51. 28165858 10.1080/10717544.2016.1261381 PMC8241190

[CIT0020] Liu R, Guo Y, Odusote G, et al. (2013a). Core-shell Fe_3_O_4_ polydopamine nanoparticles serve multipurpose as drug carrier, catalyst support and carbon adsorbent. ACS Appl Mater Interfaces 5:9167–71. 24010676 10.1021/am402585y

[CIT0021] Liu XS, Cao JM, Li H, et al. (2013b). Mussel-inspired polydopamine: a biocompatible and ultrastable coating for nanoparticles in vivo. Acs Nano 7:9384–95. 24010584 10.1021/nn404117j

[CIT0022] Liu S, Fu J, Wang M, et al. (2016). Magnetically separable and recyclable Fe 3 O 4–polydopamine hybrid hollow microsphere for highly efficient peroxidase mimetic catalysts. J Coll Interface Sci 469:69–77. 10.1016/j.jcis.2016.02.011 26871276

[CIT0023] Luo Z, Cai KY, Hu Y, et al. (2012). Cell-specific intracellular anticancer drug delivery from mesoporous silica nanoparticles with pH sensitivity. Adv Healthcare Mater 1:321–5. 10.1002/adhm.201100030 23184747

[CIT0024] Mei L, Zhang Z, Zhao L, et al. (2013). Pharmaceutical nanotechnology for oral delivery of anticancer drugs. Adv Drug Deliv Rev 65:880–90. 23220325 10.1016/j.addr.2012.11.005

[CIT0025] Narayanan S, Binulal NS, Mony U, et al. (2010). Folate targeted polymeric ‘green’ nanotherapy for cancer. Nanotechnology 21:285107. 20585151 10.1088/0957-4484/21/28/285107

[CIT0026] Park J, Brust TF, Lee HJ, et al. (2014). Polydopamine-based simple and versatile surface modification of polymeric nano drug carriers. ACS Nano 8:3347–56. 24628245 10.1021/nn405809c PMC4107448

[CIT0027] Porta F, Lamers GE, Morrhayim J, et al. (2013). Folic acid-modified mesoporous silica nanoparticles for cellular and nuclear targeted drug delivery. Adv Healthc Mater 2:281–6. 23184490 10.1002/adhm.201200176

[CIT0028] Postma A, Yan Y, Wang YJ, et al. (2009). Self-polymerization of dopamine as a versatile and robust technique to prepare polymer capsules. Chem Mater 21:3042–4.

[CIT0029] Puligujja P, Balkundi SS, Kendrick LM, et al. (2015). Pharmacodynamics of long-acting folic acid-receptor targeted ritonavir-boosted atazanavir nanoformulations. Biomaterials 41:141–50. 25522973 10.1016/j.biomaterials.2014.11.012 PMC4272445

[CIT0030] Shaw TK, Mandal D, Dey G, et al. (2017). Successful delivery of docetaxel to rat brain using experimentally developed nanoliposome: a treatment strategy for brain tumor. Drug Deliv 24:346–57. 28165821 10.1080/10717544.2016.1253798 PMC8240984

[CIT0031] Song Q, Chuan XX, Chen BL, et al. (2016). A smart tumor targeting peptide-drug conjugate, pHLIP-SS-DOX: synthesis and cellular uptake on MCF-7 and MCF-7/Adr cells. Drug Deliv 23:1734–46. 25853477 10.3109/10717544.2015.1028601

[CIT0032] Su J, Chen F, Cryns VL, Messersmith PB. (2011). Catechol polymers for pH-responsive, targeted drug delivery to cancer cells. J Am Chem Soc 133:11850–3. 21751810 10.1021/ja203077x PMC3149454

[CIT0033] Takahashi M, Yoshino T, Matsunaga T. (2010). Surface modification of magnetic nanoparticles using asparagines-serine polypeptide designed to control interactions with cell surfaces. Biomaterials 31:4952–7. 20363023 10.1016/j.biomaterials.2010.02.048

[CIT0034] Tao W, Zeng X, Liu T, et al. (2013). Docetaxel-loaded nanoparticles based on star-shaped mannitol-core PLGA-TPGS diblock copolymer for breast cancer therapy. Acta Biomater 9:8910–20. 23816645 10.1016/j.actbio.2013.06.034

[CIT0035] Tao W, Zeng X, Zhang J, et al. (2014). Synthesis of cholic acid-core poly (ε-caprolactone-ran-lactide)-b-poly (ethylene glycol) 1000 random copolymer as a chemotherapeutic nanocarrier for liver cancer treatment. Biomater Sci 2:1262–74. 32481897 10.1039/c4bm00134f

[CIT0036] Tao W, Zeng XW, Wu J, et al. (2016). Polydopamine-based surface modification of novel nanoparticle-aptamer bioconjugates for in vivo breast cancer targeting and enhanced therapeutic effects. Theranostics 6:470–84. 26941841 10.7150/thno.14184 PMC4775858

[CIT0037] Torchilin V. (2011). Tumor delivery of macromolecular drugs based on the EPR effect. Adv Drug Deliv Rev 63:131–5. 20304019 10.1016/j.addr.2010.03.011

[CIT0038] Ulbrich K, Hola K, Subr V, et al. (2016). Targeted drug delivery with polymers and magnetic nanoparticles: covalent and noncovalent approaches, release control, and clinical studies. Chem Rev 116:5338–431. 27109701 10.1021/acs.chemrev.5b00589

[CIT0039] Wang C, Cheng L, Liu Z. (2011). Drug delivery with upconversion nanoparticles for multi-functional targeted cancer cell imaging and therapy. Biomaterials 32:1110–20. 20965564 10.1016/j.biomaterials.2010.09.069

[CIT0040] Wang T, Zhu D, Liu G, et al. (2015). DTX-loaded star-shaped TAPP-PLA-b-TPGS nanoparticles for cancer chemical and photodynamic combination therapy. RSC Adv 5:50617–27.

[CIT0041] Yang B, Ni X, Chen L, et al. (2017). Honokiol-loaded polymeric nanoparticles: an active targeting drug delivery system for the treatment of nasopharyngeal carcinoma. Drug Deliv 24:660–9. 28368206 10.1080/10717544.2017.1303854 PMC8241046

[CIT0042] Yu F, Ao M, Zheng X, et al. (2017). PEG-lipid-PLGA hybrid nanoparticles loaded with berberine-phospholipid complex to facilitate the oral delivery efficiency. Drug Deliv 24:825–33. 28509588 10.1080/10717544.2017.1321062 PMC8241132

[CIT0043] Zeng X, Liu G, Tao W, et al. (2017). A drug-self-gated mesoporous antitumor nanoplatform based on pH-sensitive dynamic covalent bond. Adv Funct Mater 27:1605985.

[CIT0044] Zeng XW, Tao W, Mei L, et al. (2013). Cholic acid-functionalized nanoparticles of star-shaped PLGA-vitamin E TPGS copolymer for docetaxel delivery to cervical cancer. Biomaterials 34:6058–67. 23694904 10.1016/j.biomaterials.2013.04.052

[CIT0045] Zeng XW, Tao W, Wang ZY, et al. (2015). Docetaxel-loaded nanoparticles of dendritic amphiphilic block copolymer H40-PLA-b-TPGS for cancer treatment. Part Part Syst Charact 32:112–22.

[CIT0046] Zhang C, Zhang Z, Zhao LQ. (2016). Folate-decorated poly(3-hydroxybutyrate-co-3-hydroxyoctanoate) nanoparticles for targeting delivery: optimization and in vivo antitumor activity. Drug Deliv 23:1830–7. 26652055 10.3109/10717544.2015.1122675

[CIT0047] Zhang X, Liang X, Gu J, et al. (2017). Investigation and intervention of autophagy to guide cancer treatment with nanogels. Nanoscale 9:150–63. 27910983 10.1039/c6nr07866d

[CIT0048] Zhang XD, Yang Y, Liang X, et al. (2014). Enhancing therapeutic effects of docetaxel-loaded dendritic copolymer nanoparticles by co-treatment with autophagy inhibitor on breast cancer. Theranostics 4:1085–95. 25285162 10.7150/thno.9933 PMC4173759

[CIT0049] Zhao H, Chao Y, Liu J, et al. (2016). Polydopamine coated single-walled carbon nanotubes as a versatile platform with radionuclide labeling for multimodal tumor imaging and therapy. Theranostics 6:1833. 27570554 10.7150/thno.16047 PMC4997240

[CIT0050] Zhou K, Wang Y, Huang X, et al. (2011). Tunable, ultrasensitive pH-responsive nanoparticles targeting specific endocytic organelles in living cells. Angew Chem Int Ed Engl 50:6109–14. 21495146 10.1002/anie.201100884 PMC3438661

[CIT0051] Zhu D, Tao W, Zhang H, et al. (2016). Docetaxel (DTX)-loaded polydopamine-modified TPGS-PLA nanoparticles as a targeted drug delivery system for the treatment of liver cancer. Acta Biomater 30:144–54. 26602819 10.1016/j.actbio.2015.11.031

[CIT0052] Zhu H, Chen H, Zeng X, et al. (2014). Co-delivery of chemotherapeutic drugs with vitamin E TPGS by porous PLGA nanoparticles for enhanced chemotherapy against multi-drug resistance. Biomaterials 35:2391–400. 24360574 10.1016/j.biomaterials.2013.11.086

