# Supplementary material for: Co-delivery of docetaxel and bortezomib based on a targeting nanoplatform for enhancing cancer chemotherapy effects
Source: Drug Deliv. 2017 Aug 8;24(1):1124–38. doi: 10.1080/10717544.2017.1362677 (PMC8241102; doi:10.1080/10717544.2017.1362677)
Supplement: IDRD_Zeng_et_al_Supplemental_Content.docx [file IDRD_A_1362677_SM9413.docx]

***Supplementary Information***

**Co-delivery of docetaxel and bortezomib based on a targeting nanoplatform for enhancing cancer chemotherapy effects**

Junpeng Nie^a,c,#^, Wei Cheng^c,#^, Yunmei Peng^a,c,#^, Gan Liu^b^, Yuhan Chen^d^, Xusheng Wang^a,c^, Chaoyu Liang^c^, Wei Tao^e^, Yinping Wei^c^, Xiaowei Zeng^a,c,*^, Lin Mei^b,c,*^

*^a^School of Life Sciences, Tsinghua University, Beijing 100084, PR China;*

*^b^School of Pharmaceutical Sciences (Shenzhen), Sun Yat-sen University, Guangzhou 510275, P. R. China;*

*^c^Graduate School at Shenzhen, Tsinghua University, Shenzhen 518055, P.R. China;*

*^d^Department of Radiation Oncology, Zhongshan Hospital, Fudan University, Shanghai 200032, PR China*

*^e^Brigham and Women’s Hospital, Harvard Medical School, Boston, Massachusetts 02115, United States.*

^#^ These authors contributed equally to this work.

^*^Corresponding author. Tel./Fax: +86 75526036736.

*E-mail address:* zeng.xiaowei@sz.tsinghua.edu.cn (X. Zeng)

^*^Corresponding author. Tel./Fax: +86 2084113997

*E-mail address:* meilin7@mail.sysu.edu.cn (L. Mei)





**Figure S1.** The covalent bond of catechol and boronic acid structure in BTZ forms and remains stable at neutral and alkaline pH; however it dissociates in acid environment easily. The pH-responsive bond contributes to inhibit the activity of BTZ in neutral circulation and release free BTZ at the acid tumor sites.





**Figure S2.** The size distribution by intensity of DTX-loaded CA-PLGA@PDA-PEG/NPs.





**Figure S3.** The different loading content of DTX and BTZ under different BTZ concentration.


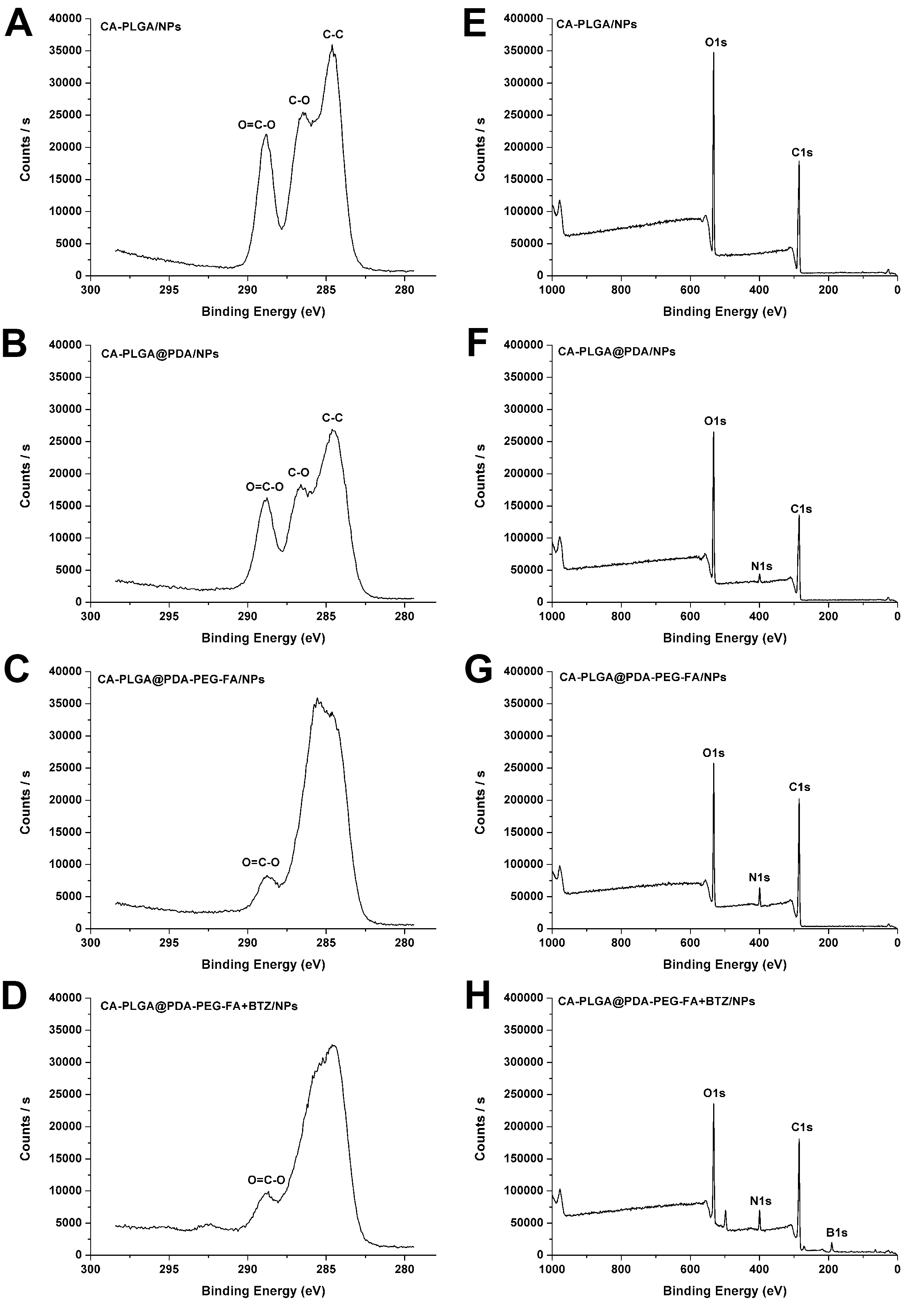


**Figure S4.** XPS spectra of NPs. (A)-(D) narrow scan of C1s peaks; (E)-(H) wide scan.





**Figure S5.** In vitro BTZ release profile of DTX-loaded CA-PLGA@PDA-PEG-FA+BTZ/NPs upon shifting of pHs from neutral to acidic condition.





**Figure S6.** Viability of MCF-7 cells cultured with drug-free CA-PLGA@PDA-PEG-FA/NPs at a NPs concentration series of 2.5, 25, 125, and 250 μg/ml for 24 h and 48 h.





**Figure S7.** DTX concentration-time profile following intravenous administration of Taxotere®, DTX-loaded CA-PLGA@PDA/NPs, DTX-loaded CA-PLGA@PDA-PEG/NPs, DTX-loaded CA-PLGA@PDA-PEG-FA/NPs and DTX-loaded CA-PLGA@PDA-PEG-FA+BTZ/NPs.





**Figure S8.** BTZ concentration-time profile following intravenous administration of BTZ and DTX-loaded CA-PLGA@PDA-PEG-FA+BTZ/NPs.
